# A novel lysin Ply691 exhibits potent bactericidal activity against *Streptococcus suis*

**DOI:** 10.3389/fvets.2025.1653748

**Published:** 2025-09-08

**Authors:** Yanhong Shang, Xinyi Li, Shihang Ren, Congyang Du, Stefan Schwarz, Chenglong Li, Xiang-Dang Du

**Affiliations:** ^1^College of Veterinary Medicine, Henan Agricultural University, Zhengzhou, China; ^2^Department of Veterinary Medicine, Centre for Infection Medicine, Institute of Microbiology and Epizootics, Freie Universität Berlin, Berlin, Germany; ^3^Veterinary Centre of Resistance Research (TZR), Freie Universität Berlin, Berlin, Germany

**Keywords:** *Streptococcus suis*, phage, lytic enzyme, lytic activity, bacteremia

## Abstract

**Introduction:**

*Streptococcus suis* represents a growing zoonotic pathogen, exacerbated by increasing antimicrobial resistance due to a widespread and often inappropriate antimicrobial use. This escalating challenge underscores the pressing need for innovative treatment strategies against streptococcal infections in pigs. In our study, we identified Ply691, a prophage-encoded lytic enzyme.

**Methods:**

The corresponding gene was identified during whole genome analysis of *S. suis* SC267. Structural domain analysis revealed that Ply691 consists of an N-terminal Amidase-5 catalytic domain, a C-terminal Glucosaminidase catalytic domain, and two centrally located CW-7-binding structural domains. In order to investigate the bactericidal potential of Ply691, an in vitro bactericidal assay was conducted using Ply691, and its bactericidal effect was evaluated by colony counting method after applying it to different strains of bacteria and at different temperatures and pH conditions. Subsequently, a mouse bacteremia model was established, and the *in vivo* bactericidal efficacy of Ply691 was evaluated by measuring the bacterial residues in the blood and different organs of mice treated with Ply691.

**Results:**

*In-vitro* antimicrobial susceptibility testing demonstrated that Ply691 exhibits potent lytic activity against 11 serotypes of *S. suis*, including serotypes 2, 3, 5, 9, 10, 12, 17, 18, 19, 29, and 30. Furthermore, Ply691 reduced the number of *S. suis* colonies by approximately 1 log within 20 min. Ply691 also displayed a broad temperature adaptability range (4°C-37°C) and remarkable alkaline tolerance (pH 7-10). In an *in vivo* murine bacteremia model, Ply691 demonstrated significant therapeutic effects. Administration of Ply691 at a dose of 2 mg per mouse by intraperitoneal injection an hour post-infection resulted in a 100% survival rate and substantially reduced the bacterial load in the blood and various organs (heart, liver, spleen, lung, kidney, and brain). Histological analysis confirmed that these organs closely resembled those of the control group.

**Discussion:**

Ply691 exhibits broad-spectrum lytic activity against *S. suis* with unique structural advantages. It demonstrates robust efficacy *in vivo* without inducing resistance, showing significant therapeutic potential for streptococcal infections.

## 1 Introduction

*Streptococcus suis* is a significant zoonotic pathogen that causes a range of diseases in both pigs and humans, including meningitis, septicemia, arthritis, endocarditis, and pneumonia in pigs, as well as meningitis, septicemia, and streptococcal toxic shock syndrome in humans (1). This pathogen not only imposes substantial economic losses on the swine industry but also poses a considerable threat to public health (2). In recent years, the treatment of *S. suis* infections in swine has become increasingly challenging due to increasing antimicrobial resistance, likely based on the long-term, extensive, and at least in part inappropriate use of antimicrobial agents (3). Consequently, there is an urgent need to explore innovative strategies for the treatment of porcine streptococcal infections.

Phage lytic enzymes can hydrolyze the peptidoglycan in bacterial cell walls, leading to bacterial lysis (4). Peptidoglycan, an essential and highly conserved component of the cell wall, makes bacteria less likely to develop resistance to lysins (5, 6). Despite the potential of lysins, isolating *S. suis*-specific phages has proven challenging, with only one potent phage, SMP, identified globally. However, SMP has a narrow lysis spectrum, targeting only two strains of *S. suis* type 2 (7). Thus, phage SMP alone does not meet the criteria for a broad-spectrum anti-*S. suis* agent.

Phage lytic enzymes, present in lysogenic phages, can be obtained by identifying lytic enzyme genes from prophage sequences in bacterial genomes. So far, prophage lytic enzymes have been reported in *S. suis*. For instance, PlySs2, a prophage lytic enzyme of *S. suis* type 2, could lyse five Gram-positive bacteria *in-vivo* and *in-vitro* (8), and Ply30 derived from the *S. suis* type 9, has lytic activity against *S. suis* and *Streptococcus equi* (9). Ly7917, Ply5218, and Ply1228 are lysases encoded by the prophages of strains of *S. suis* serotypes SS7, SS9, and SS12, respectively, also exhibit lytic activities against *S. suis* (10–12).

In this study, we identified and expressed a lysin, Ply691, derived from the *S. suis* SS10 strain SC267. Ply691 has a unique four structural domains compared to the studied conventional lysases, and has a wider temperature adaptability and strong alkali resistance than other lysases. Among them, Ply691 also showed great advantages in the determination of lysate profiles, which can effectively target 11 porcine Streptococcus serotypes, and stood out for its strong bacteriolytic activity, and also showed strong bactericidal and therapeutic effects in *in vivo* therapeutic models, Ply691 showed great potential for clinical applications. Ply691 demonstrates promising activity and warrants further preclinical evaluation.

## 2 Materials and methods

### 2.1 Strains and growth conditions

The 27 *S. suis* strains, three strains each of *Staphylococcus aureus*, *Enterococcus faecalis* strains, *Enterococcus faecium* strains, and the prokaryotic expression vector pET28a (+) used in this study were all obtained from our laboratory. *E. coli* DH5α and *E. coli* BL21(DE3) were purchased from Beijing Zoman Biotechnology Co., Ltd. All *S. suis* cultures were grown in Todd-Hewitt broth (THB; Haibo Bio, Qingdao, China), with the addition of 5% calf serum (Zhejiang Tianhang Bio-Reagent Co., Ltd.). *S. aureus*, *E. faecalis*, and *E. faecium* were cultured in brain heart infusion broth (BHI; Haibo Biotechnology, Qingdao, China). *E. coli* DH5α and *E. coli* BL21(DE3) were cultured in lactose broth (LB; Haibo Biotechnology, Qingdao, China). All bacterial cultures were incubated at 37 °C with shaking at 180 rpm overnight until the OD_600_ reached 0.8 for use in experiments. When using competent cells containing the prokaryotic expression vector pET28a (+), 50 μg/mL kanamycin was added.

### 2.2 Whole-genome sequencing and analysis of *Streptococcus suis* SC267

The assembled *S. suis* SC267 genome was analyzed with Glimmer 3.0 for open reading frames (ORFs) prediction to identify potential lysin genes. Potential lysin gene sequences were identified by analyzing the fully sequenced genome of *S. suis* SC267. Amino acid sequences were compared using BLAST from NCBI to determine similarity with putative lytic enzymes. Structural domains were analyzed using online tools, including Pfam^1^ and CDD.^2^

### 2.3 Prokaryotic expression of Ply691

Based on the sequence of Ply691 gene in SC267 sequenced in the laboratory, primers were designed by primer software, and BamHI and SalI cleavage sites were added to the front of Ply691-F and Ply691-R, respectively. Ply691-F: cgcggatccATGGGGAGTTAATATT GAAACTG; Ply691-R: cgcggtcgacTAATCATACTGGTTTTTT CTCCAGTTT. The expression vector pET-28a and ORF691 gene fragments were double digested with SalI and BamHI, and ligated with T4 ligase at 4 °C overnight. The ligated productivities were transferred into *E. coli* DH 5 α receptor cells, and the positive clones were identified by double digestion, specific PCR and sequencing. The recombinant plasmid pET28a(+)-Ply691 was chemically transformed into *E. coli* BL21(DE3) competent cells. This recipient cells were cultured in LB broth supplemented with kanamycin (50 μg/mL) at 37 °C and 180 rpm until the optical density reached OD_600_ = 0.6–0.8. Subsequently, induction was carried out using IPTG (1.0 mM) at 16 °C and 180 rpm for 14 h. The culture was then centrifuged at 4 °C and 8,000 × g for 5 min. The resulting pellets were resuspended in sterile PBS, sonicated, and centrifuged to collect the supernatant containing the crude Ply691 lysin extract. After filtration through a 0.22 μm membrane, the extract was purified using Ni-TED agarose resin and concentrated by ultrafiltration. The size and expression of the recombinant protein were confirmed using SDS-PAGE.

### 2.4 *In-vitro* cleavage activity of Ply691

*Streptococcus suis* SC267 was cultured to the logarithmic phase (OD_600_ = 0.6–0.8), then centrifuged to collect pellets. The pellets were washed three times with sterile PBS buffer and resuspended in PBS. Purified Ply691 protein was added to achieve final concentrations of 50 μg/mL, 100 μg/mL, and 200 μg/mL in the bacterial solutions. For the control group, an equal volume of sterile binding buffer solution was used. All four groups were incubated at 37 °C for 1 h, with samples taken at 20 min intervals for colony counting. The experiment was repeated three times to ensure the reliability of the results.

*S. suis* SC267 was cultured to the logarithmic phase and spread onto THB plates supplemented with 5% fetal bovine serum. After the bacterial solution dried, 10 μL of Ply691 lysin (200 μg/mL) was applied dropwise. The plates were then incubated overnight at 37 °C. Single colonies from the edge of the inhibition zone were subsequently selected and incubated in THB medium with 5% fetal bovine serum overnight. This process was repeated for 12 generations to assess the bactericidal effect of Ply691 lysin on each SC267 generation.

### 2.5 Cleavage spectrum of Ply691

Twenty seven *S. suis* strains, along with three strains each of *S. aureus*, *E. faecalis*, and *E. faecium* were cultured to logarithmic growth, washed thrice with sterile PBS solution and resuspended. Subsequently, the lysin Ply691 was added to reach 200 μg/mL, and the control received an equal amount of sterile binding buffer. The cultures were then, incubated for 1 h at 37 °C and followed by colony counting. This experiment was repeated thrice for consistency.

### 2.6 Temperature and pH stability of the lytic enzyme Ply691

SC267, cultured to logarithmic phase, was washed three times with sterile PBS and resuspended. The bacterial suspension was mixed with the lytic enzyme Ply691 to obtain final concentration of 200 μg/mL, while the control group received an equal amount of sterile Binding buffer. These samples were incubated at temperatures of 4 °C, 12 °C, 25 °C, 37 °C, and 45 °C for 1 h, respectively. The colony counts were determined using the fold dilution method. The bactericidal activity of Ply691 against *S. suis* SC267 was also assessed under various pH conditions using Binding buffer (pH 4, 5, 6, 7, 8, 9, 10), with colony counting performed for each pH. Equal volumes of sterile Binding buffer with different pH were used in the control group, and colony counts were performed., and the colonies were counted. This experiment was repeated three times (13).

### 2.7 Ethics statement

Female BALB/c mice, weighing 16–18 grams, were obtained from Huaxing Animal Farm in Huiji District, Zhengzhou, China. In this experiment, euthanasia was performed by intraperitoneal injection of sodium pentobarbital (100 mg/kg), and tissue sampling was conducted after confirming cardiac arrest. During the experiment, indicators such as mouse body temperature, weight, and activity level were monitored. If a mouse exhibited hypothermia (<30 °C), severe motor dysfunction, or weight loss >20%, euthanasia was performed prematurely. All experimental procedures were approved by the Institutional Animal Care and Use Committee (IACUC) of Henan Agricultural University (approval no. SY202010032) and conducted in strict accordance with the Regulations on the Administration of Laboratory Animals, as stipulated by the State Council of the People’s Republic of China.

### 2.8 Determination of minimum lethal dose

*Streptococcus suis* SC267, cultured to the logarithmic growth stage, was washed three times with sterile PBS and resuspended to yield concentrations of 1.0 × 10^9^ Colony-Forming Units per milliliter (CFU/mL), 1.0 × 10^8^ CFU/mL, 1.0 × 10^7^ CFU/mL, and 1.0 × 10^6^ CFU/mL, respectively. Groups of mice (n = 6 per group) were intraperitoneally injected with the respective doses of either 1.0 × 10^9^, 1.0 × 10^8^, 1.0 × 10^7^ or 1.0 × 10^6^ CFU per mouse. The survival rate of each group was monitored over a 7-day period. The dose causing mortality in all mice was determined as minimum lethal dose (MLD) (14).

### 2.9 Determination of the protective rate of Ply691 against bacteremia in mice

An infection dose of 2 × MLD of porcine *S. suis* SC267 was administered to induce a systemic infection. Blood was collected from the tail vein every 1 h within 7 h after the attack, and the number of colonies in peripheral blood was measured by double dilution. This was confirmed when *in-vivo* blood colony counts reached 10^6^ CFU/mL. Treatment commenced at this point with Ply691 doses of 2 mg and 1.5 mg per mouse via intraperitoneal injection, while the control group received an equal volume of sterile PBS (*n* = 6 mice per group). Survival rates were recorded for each group over a 7-day period (15).

To assess the safety of the lytic enzyme Ply691 in mice, one group was administered a high dose (2 mg/mouse) via a single intraperitoneal injection, while the control group received an equal amount of sterile saline (*n* = 6 mice per group). The health status of the mice in each group was monitored over 7 days using a rating scale ranging from 5 to 0. Health status is normal, with no obvious symptoms, scored as 5; disheveled fur and sluggish movement are defined as mild disease, scored as 4; lethargy and kyphosis are defined as moderate disease, scored as 3; the presence of the above symptoms along with exudate around the eyes is defined as severe disease, scored as 2; near-death condition is scored as 1; death is scored as 0. Each point represents the health score of a single mouse.

### 2.10 Therapeutic effect of Ply691 on bacteremia in mice

To assess the bacterial load in mice following SC267 infection, two groups of mice (*n* = 10) were treated with either Ply691 (2 mg/mouse) or an equal volume of sterile PBS solution an hour post infectionem (p.i.). At various time intervals (1, 2, 3, 4, 5, 6, 12, 24, 36, and 48 h p.i.), three mice from each group were randomly selected for tail vein blood collection. In addition, the remaining seven mice in each group were sacrificed at specific time points (1, 12, and 24 h), and heart, liver, spleen, lung, kidney, and brain tissues were harvested, weighed, and processed along with the blood samples to measure bacterial load.

For histopathological analysis, At 24 h, 48 h, and 72 h after infection, 3 mice were randomly selected in each group and tissues from these organs were collected, fixed in 4% paraformaldehyde, processed into paraffin sections, and examined using a fully automated biomicroscope for scanning, imaging, and analysis.

### 2.11 Statistical analysis

All statistical analyses were conducted using Prism software (GraphPad). Data were analyzed using one-way analysis of variance (ANOVA) followed by Tukey’s *post-hoc* test for multiple comparisons. *p* values < 0.05 were considered statistically significant.

## 3 Results

### 3.1 Structural domain analysis and prokaryotic expression of Ply691

**Figure 1:**
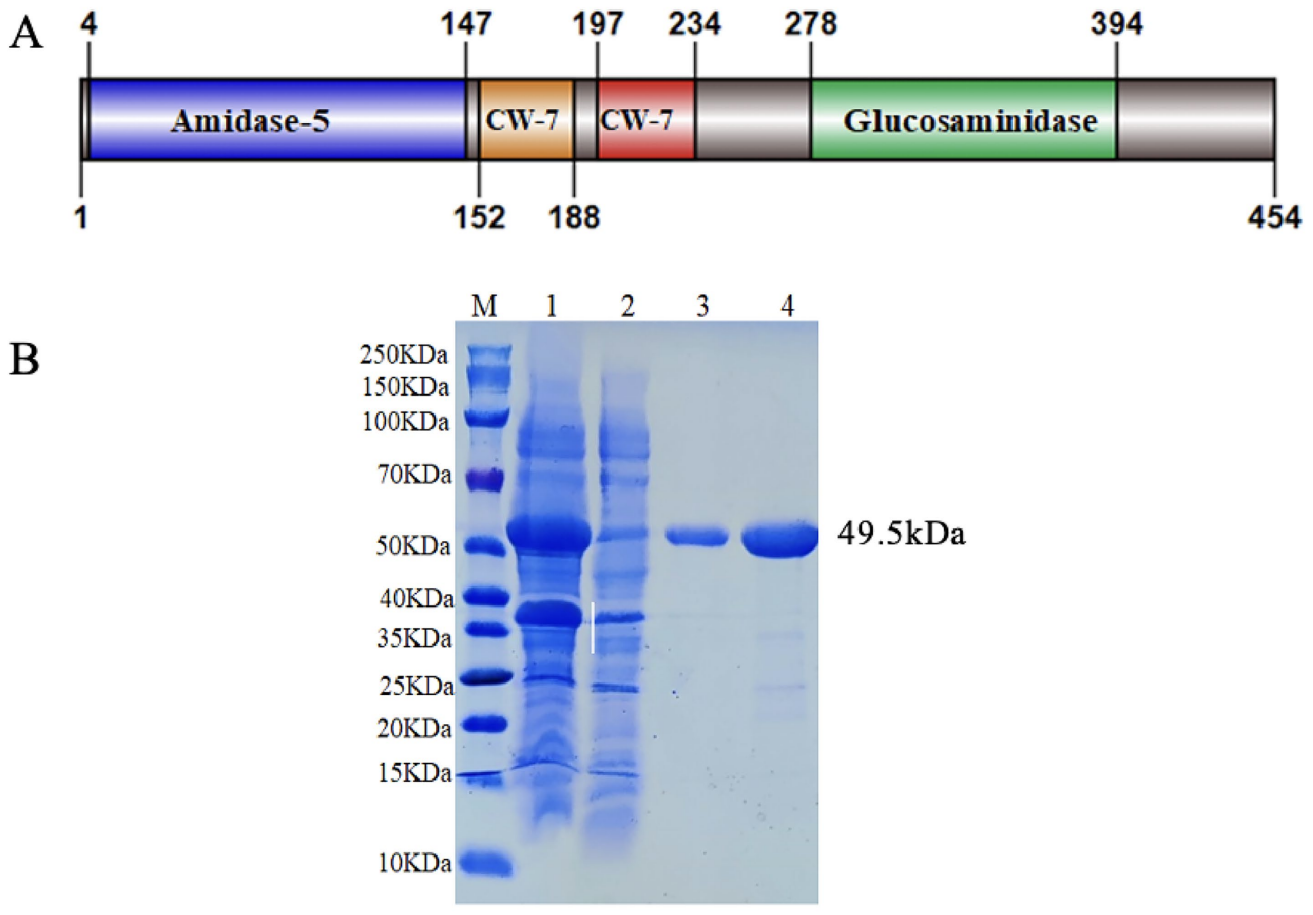
Domain organization and purified SDS-PAGE electrophoresis pattern of Ply691. **(A)** Domain organization of Ply691. Ply691contains four domains: the amidase-5 domain (blue, residues 4–147), and two cell wall binding domains composed of CW-7 repeats (orange and red, residues 152–234), the CHAP domain (blue, residues 278–394). **(B)** Purified SDS-PAGE electrophoresis pattern of Ply691. Lanes: (M) size marker, (1) Unpurified protein supernatant, (2) flow-through fluid, (3–4) Purified Ply691.

Through ORFs analysis of *S. suis* SC267, ORF691 was predicted to encode a cleavage enzyme. Therefore, we named it Ply691. Through NCBI BLAST comparison of Ply691, Ply691 exhibits high aa sequence homology of 71 and 65.4% with *S. suis* lysins LySMP and Ply1228, respectively, but less than 25% homology with other *S. suis* lysins, such as PlySs2, Ply30, Ply5218, and Ly7917. Structural domain analysis in the NCBI database indicated that Ply691 comprises four structural domains: a N-terminal Amidase-5, belonging to the NLPC-P60 superfamily of peptidoglycan hydrolases (4–147 aa), which acts as a CHAP (cysteine-histidine-dependent amide hydrolases/endopeptidases) catalytic domain; a C-terminal Glucosaminidase, serving as another catalytic structural domain (278–394 aa); and two centrally located CW-7 binding structural domains (152–188 aa and 197–234 aa) (Figure 1A).

### 3.2 Recombinant expression of ply 691

The expression strain BL21(DE3)-pET28a(+)-Ply691 was constructed and the correct amino acid sequence of Ply691 on the plasmid was confirmed by sequencing. After induction and purification using a nickel column, a band with a molecular weight of 49.5 kDa was observed (Figure 1B).

### 3.3 *In-vitro* cleavage activity of Ply691

**Figure 2:**
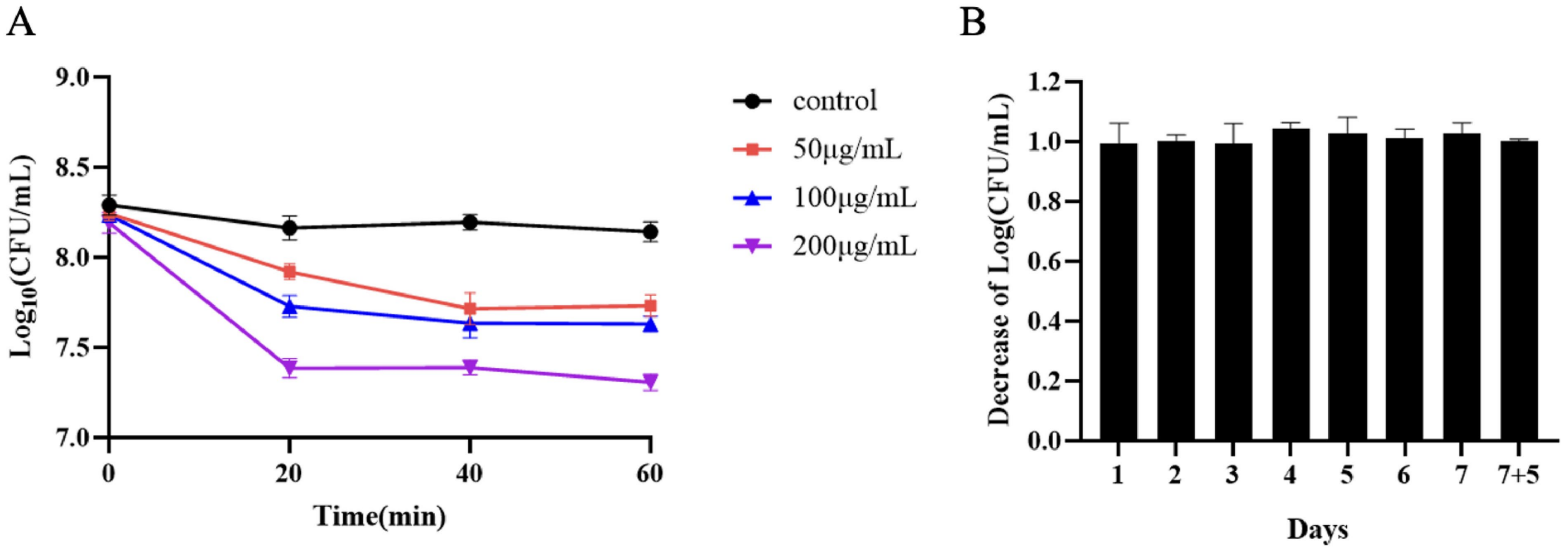
*In-vitro* cleavage activity of Ply691. **(A)** Bacterial residue levels at 20-min intervals within 1 h of exposure to different concentrations of the protease Ply691 on SC267. As a control, *S. suis* SC267 was treated with an equivalent quantity of PBS buffer. **(B)** The bactericidal activity of Ply691 against the 12th generation of bacteria SC267. Log10 (CFU/mL) decrease in the *S. suis* SC267 culture over many generations was used to evaluate whether the sensitivity of bacteria to Ply691 was changed. The values are the means ± SDs (*n* = 3).

During the logarithmic growth phase of *S. suis* SC267, the addition of Ply691 at concentrations of 200 μg/mL resulted in approximately a 1-log reduction in bacterial count. At lower concentrations of 50 μg/mL and 100 μg/mL, Ply691 exhibited a similar bactericidal effect, reducing bacterial counts by about 0.5 logs (Figure 2A). The maximum bactericidal effect was observed when Ply691 was in contact with *S. suis* SC267 for 20 min, with no further reduction in bacterial count observed thereafter.

Over 12 generations, Ply691 consistently demonstrated bactericidal effects against *S. suis* SC267 without any significant changes (Figure 2B), suggesting that the bacteria did not develop resistance to Ply691 within this timeframe.

### 3.4 Cleavage spectrum of Ply691

**Figure 3:**
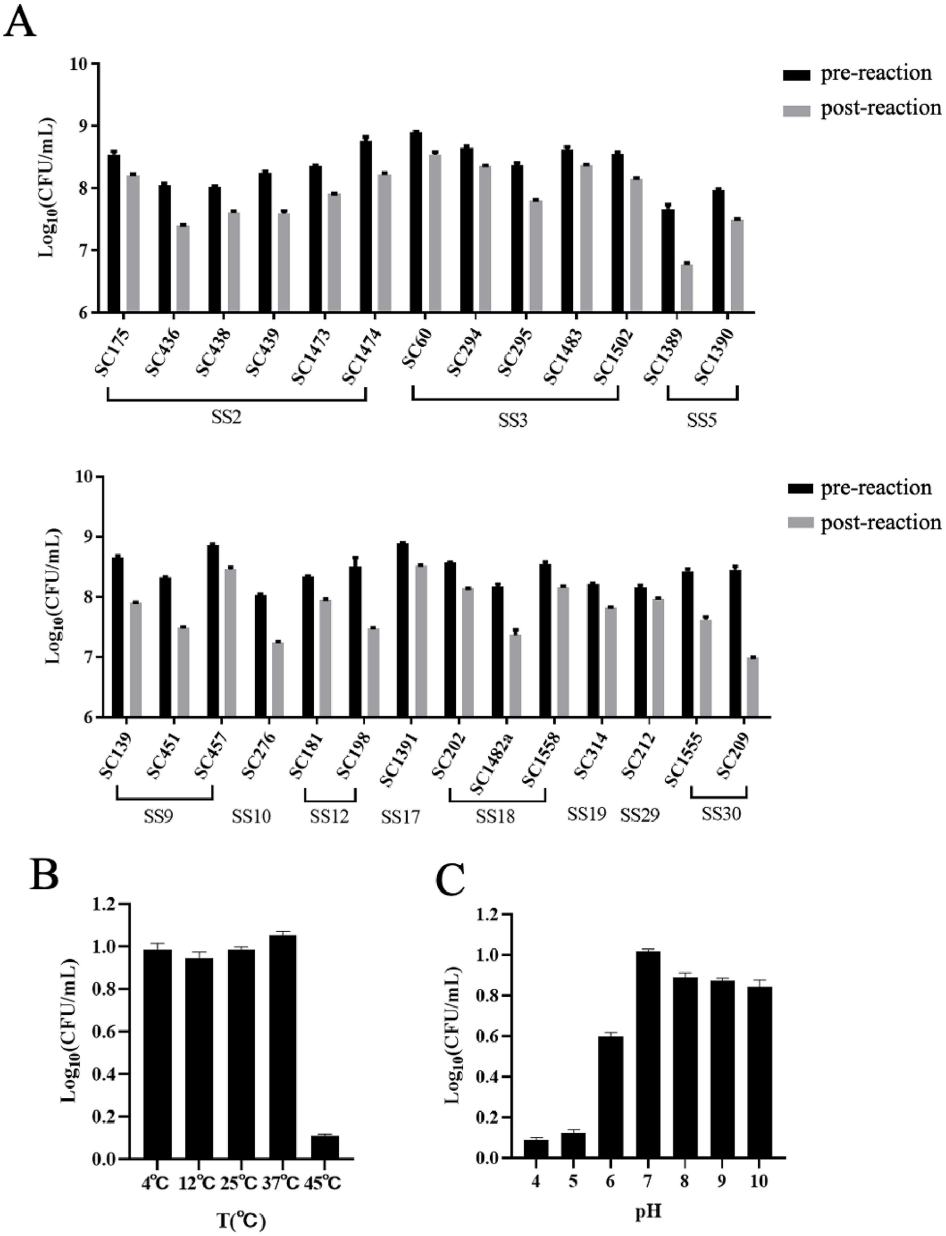
Lytic spectrum of Ply691 and effect of pH value and temperature on the activity of Ply691. **(A)** Lytic activities of Ply691 against different serotypes of *S. suis*, the difference between the number of colonies after incubation with Ply691 (post-reactio) and the initial number of colonies (pre-reaction) is the lysing activity of the lytic enzyme Ply691 against different strains of bacteria. **(B)** Effect of different temperature (4 °C, 12 °C, 25 °C, 37 °C, 45 °C) on the activity of lysin Ply691. The bactericidal activity of lyase Ply691 at different temperatures was assessed by measuring the logarithmic value (Log10) of colony-forming units (CFU/mL) of the *S. suis* SC267 strain. **(C)** Effect of different pH (4, 5, 6, 7, 8, 9, 10) value on the activity of lysin Ply691. The bactericidal activity of Ply691 at different pH levels was assessed by measuring the logarithmic value (Log10) of colony-forming units (CFU/mL) of the *S. suis* SC267 strain.

Ply691 demonstrates a broad spectrum of lytic activity against *S. suis*, effectively lysing all 11 serotypes, including types 2, 3, 5, 9, 10, 12, 17, 18, 19, 29, and 30 (Figure 3A). However, Ply691 showed no lytic effect on *S. aureus*, *E. faecalis*, or *E. faecium* (data not shown in the figure).

### 3.5 Temperature and pH stability of Ply691

At 45 °C, Ply691 displayed significantly reduced lytic activity. However, temperatures ranging from 4 °C to 37 °C had minimal impact, with Ply691 maintaining stable lytic activity within this range. The optimal temperature for Ply691 activity was found to be 37 °C (Figure 3B). Under alkaline conditions (pH 7–10), Ply691 exhibited consistent lysin activity, reaching its peak at pH 7 (Figure 3C).

### 3.6 MLD determination

**Figure 4:**
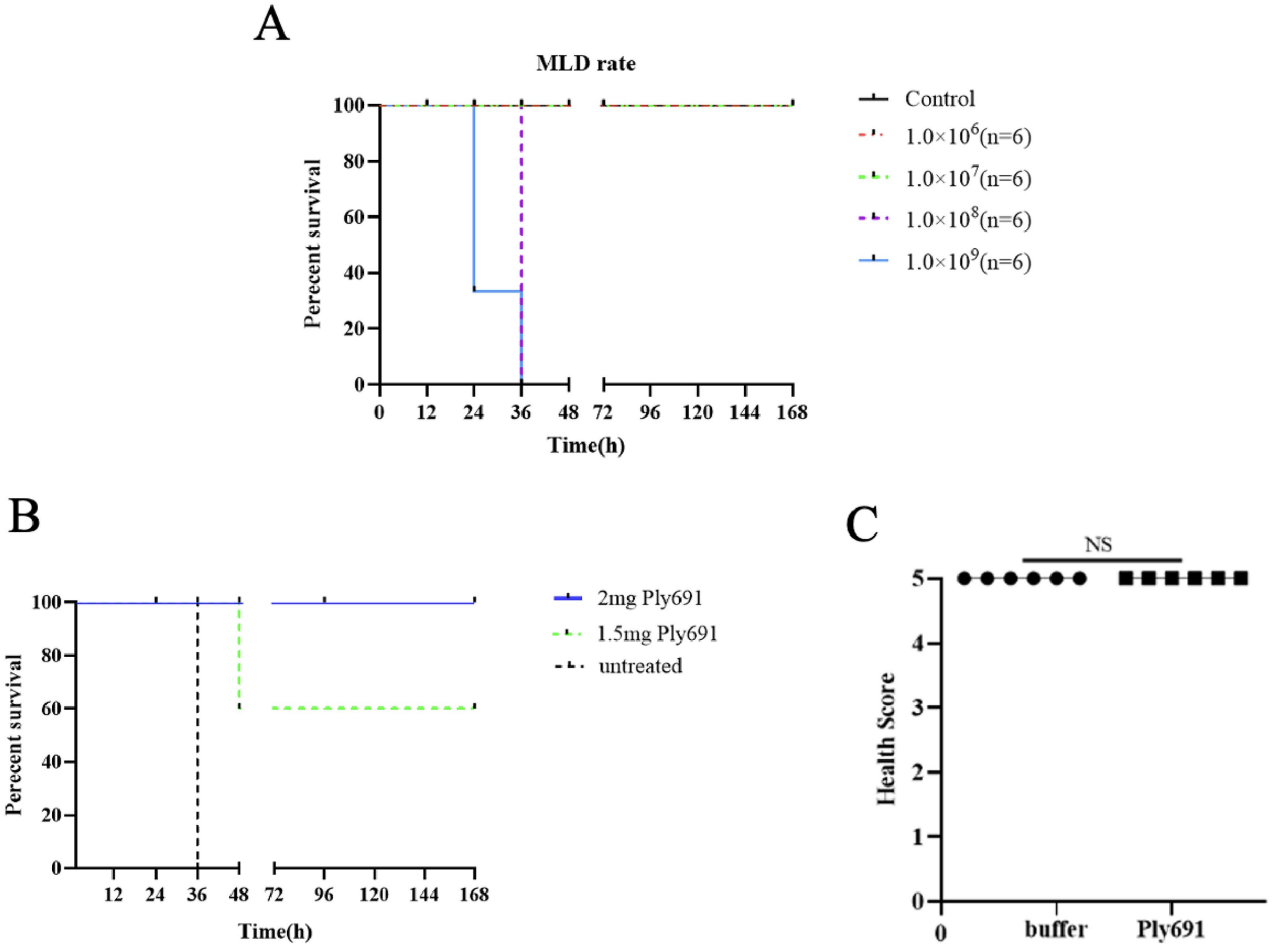
Therapeutic effects of Ply691. **(A)** Survival of mice after challenge at different concentrations of SC267. SC267 was applied to mice (6 mice per group) at four different concentrations (1.0 × 10^9^ CFU/mL, 1.0 × 10^8^ CFU/mL, 1.0 × 10^7^ CFU/mL, 1.0 × 10^6^ CFU/mL). The last group of mice was treated with PBS as a control. Determine the minimum lethal dose by testing the survival rate of mice over a period of 7 days. **(B)** Survival of mice after treatment with different concentrations of Ply691. All mice were injected with 2 times the minimum lethal dose of SC267 to induce systemic infection. Two groups of mice were administered intraperitoneal injections at doses of 2 mg/mouse and 1.5 mg/mouse, respectively (6 mice per group), while the last group received sterile PBS as a control. The survival rate of mice was monitored over a 7-day period to determine the protective efficacy of the lysozyme Ply691 against bacteremia in mice. **(C)** Safety of Ply691 in mice. Two groups of mice (6 mice per group) were treated with 200 μg/mouse Ply691 or equal amount of sterile saline. The health status of the mice was scored on a scale of 0–5. The safety of Ply691 was assessed by comparing the scores of the two groups. N. S., not significant.

Mice injected with *S. suis* SC267 at doses of 1.0 × 10^8^ CFU/mouse or higher died within 36 h, indicating the presence of significant bacteremia. Consequently, the MLD for mice was determined to be 1.0 × 10^8^ CFU/mouse (Figure 4A).

### 3.7 Determination of the protective rate of Ply691 against bacteremia in mice

To evaluate Ply691’s protective efficacy against acute bacteremia induced by *S. suis* SC267, mice received an intraperitoneal injection of 2 × MLD (2.0 × 10^8^ CFU/mouse) of SC267. This elevated their blood bacterial load to 3.5 × 10^6^ CFU/mL after 1 h (Figure 4B), indicating systemic infection, with untreated mice died within 36 h (Figure 4B). Subsequently, various doses of Ply691 were administered. As depicted in Figure 4B. a dosage of 2 mg/mouse Ply691 achieved a 100% survival rate within 7 days, while a 1.5 mg/mouse dose resulted in 60% survival. Moreover, a single 2 mg intraperitoneal injection of Ply691, compared to an equal volume of sterile saline, did not induce apparent toxic effects in mice, as evidenced by their appearance and behavior remaining comparable to those in the control (Figure 4C).

### 3.8 Therapeutic effect of Ply691 on bacteremia in mice

**Figure 5:**
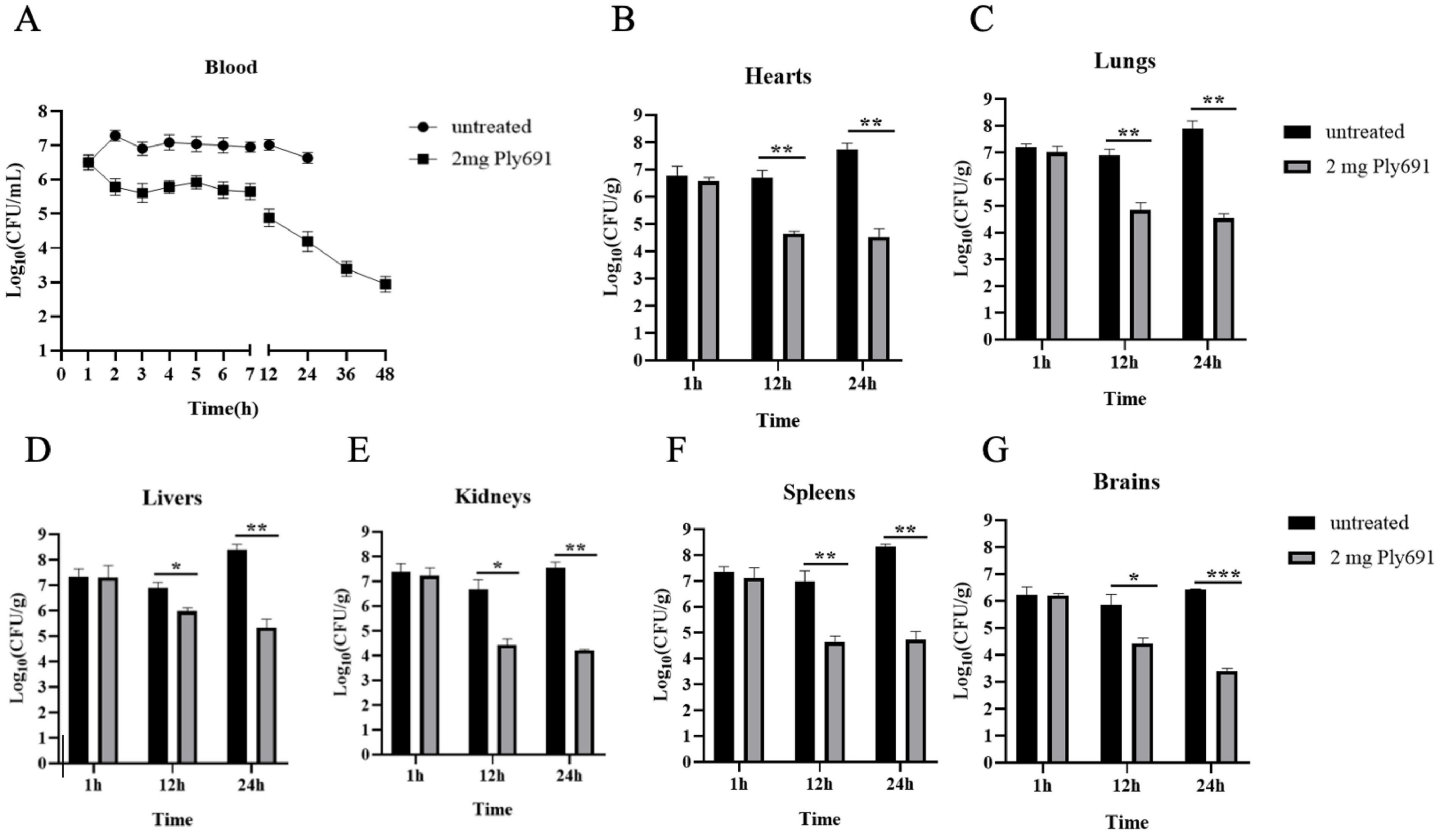
The therapeutic effect of Ply691 on bacteremia in mice. **(A)** Bacterial load in blood. Two groups of mice (10 mice per group) were treated with Ply691 (2 mg/mouse) and an equal volume of sterile PBS, respectively, 1 h after infection with SC267. Blood samples were collected from the tail veins of randomly selected mice (*n* = 3) at different time points, and bacterial load was determined by colony count. Bacterial load in the heart **(B)**, lungs **(C)**, liver **(D)**, kidneys **(E)**, spleen **(F)**, and brain **(G)**. At different time points (1 h, 12 h, and 24 h) after infection with SC267, the hearts, lungs, livers, kidneys, spleens, and brains of euthanized mice were removed, weighed, homogenized, diluted, and subjected to colony counting to determine bacterial load in different tissues (*n* = 7). The data presented are the mean ± standard deviation of three replicate experiments. *, **, and *** indicate significant differences at *p* < 0.05, *p* < 0.01, and *p* < 0.001, respectively.

The therapeutic efficacy of Ply691 was evaluated by assessing the bacterial load in both peripheral blood and various organs (heart, liver, spleen, lungs, kidneys, and brain). In mice treated with PBS, the bacterial load in the blood steadily rose to 10^7^ CFU/mL (Figure 5A). In contrast, Ply691-treated mice displayed no significant change in blood bacterial load for the initial 7 h. However, a notable decline was observed starting at 12 h, reaching 1.2 × 10^3^ CFU/mL by 48 h (Figure 5A).

At 24 h post-SC267 infection, the PBS-treated group exhibited the highest bacterial loads in the heart, liver, lung, kidney, spleen, and brain. Yet, by 12 h, the Ply691-treated group showed significantly reduced bacterial loads in these organs compared to the PBS-treated group (Figures 5B–G). Furthermore, bacteria that remained unaffected by Ply691 remained sensitive to it at all measured time points, indicating that SC267 did not develop resistance to Ply691 (data not shown).

**Figure 6:**
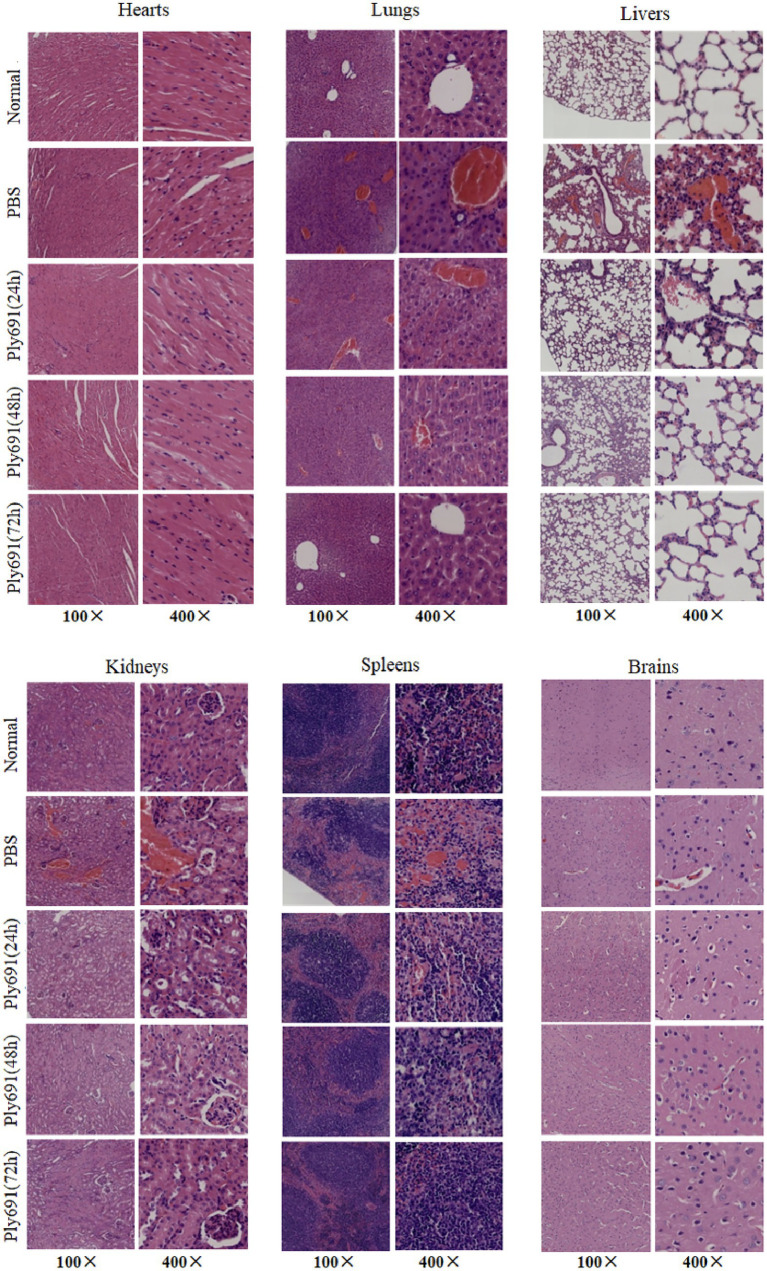
Histopathology of different tissues. Mice infected with SC267 were randomly divided into three groups: those treated with Ply691, those treated with PBS, and a control group. Euthanasia was performed at different time points post-treatment (24 h, 48 h, and 72 h). Tissue samples from the heart, liver, lungs, kidneys, spleen, and brain were collected for sectioning and hematoxylin and eosin (HE) staining, with objective lens magnifications of 10× and 40×. The therapeutic effect of Ply691 on mouse bacteremia was evaluated by comparing the pathological changes between the experimental group and the control group.

Histopathological analysis (Figure 6) revealed progressive pathological changes in untreated mice, including lung congestion, hemorrhage, alveolar capillary dilation and congestion, thickening of alveolar walls, and infiltration of inflammatory cells in the alveolar lumens. However, by 48 h post-treatment, the majority of lung alveolar structures appeared normal, and by 72 h, no significant pathological changes were evident compared to the control group.

In untreated mice, the spleens exhibited venous sinus congestion and hemorrhage, with areas of hemorrhage containing necrotic lymphocytes and some neutrophil infiltration. These changes were comparable to the PBS-treated control group at 48 h. As the disease progressed in the PBS-treated group, liver alterations included erythrocyte sludging in the interhepatic cords, vascular congestion, along with mild inflammatory infiltration in the heart and kidney interstitium, and liquefactive necrosis in brain tissue. By 72 h post-treatment, these pathological changes resembled those observed in the control group.

Conversely, in the Ply691 treatment group, histopathological damage gradually diminished over the 24-, 48-, and 72-h periods. By 72 h, the heart, liver, lung, kidney, spleen, and brain appeared similar to the control group. Furthermore, the Ply691 treatment group closely resembled the untreated group in terms of histopathological findings.

## 4 Discussion

In this study, our whole genome sequencing analysis revealed a lysin encoded by the lysogenic phage of *S. suis* SS10 strain SC267, which we named Ply691. Comparing Ply691 to other lysins in the NCBI database, we found that Ply691 consists of a CHAP (cysteine-histidine-dependent amide hydrolases/endopeptidases) structural domain at its N-terminus and a Glucosaminidase structural domain at its C-terminus, accompanied by two central CW-7 binding domains. The presence of two CW-7 binding domains further enhances cell wall targeting, potentially increasing binding avidity and specificity compared to lysins with a single binding module. The N-terminal Amidase-5 belongs to the NLPC-P60 superfamily and serves as a catalytic CHAP domain (16, 17). The combination of a CHAP domain and glucosaminidase domain in Ply691 likely confers synergistic peptidoglycan degradation, as CHAP domains cleave peptide cross-links while glucosaminidases hydrolyze glycan strands.

Unlike conventional lysins that possess dual structural domains (18), Ply691 stands out with its unique set of four structural domains. For example, Ply5218 features an Amidase-2 amidase catalytic structural domain along with an SH3b-binding structural domain (11). Similarly, both Ply30 and PlySs2 are equipped with an N-terminal CHAP catalytic structural domain and a C-terminal SH3b-binding structural domain (9, 19). Consequently, Ply691 exhibits substantial differences in both its amino acid sequence and structural domain composition compared to other known *S. suis* lysins.

Ply691 exhibits robust lytic activity against *S. suis* SC267 over a broad range of conditions. Specifically, it remains effective when incubated for 1 h at temperatures ranging from 4 °C to 37 °C and pH levels from 7 to 10. This is in contrast to other lytic enzymes: (1) LysGH15 from *S. aureus* displays lytic activity exclusively within a pH range of 5–8, and its effectiveness drops considerably below 20 °C (20); (2) Ply30 shows activity only at pH levels above 6, with no information available regarding its temperature stability (9); (3) PlySs2 from porcine *S. suis* SS2 retains its cleavage activity for a mere 15 min at 4 °C (21). In comparison, Ply691’s wide temperature adaptability and robust alkaline tolerance set it apart, making it a promising candidate for various applications.

Ply691 displays an expansive cleavage spectrum, effectively targeting 11 serotypes of *S. suis* (serotypes 2, 3, 5, 9, 10, 12, 17, 18, 19, 29, and 30). In comparison, other lysins have more constrained activities: (1) Ply5218, a lysin derived from *S. suis* SS9, has been reported to lyse only *S. suis* serotype 2 (11); (2) Ly7917, a lysin derived from *S. suis* SS7, has lysing activity against *S. suis* serotypes 1, 2, 7, and 9 (10); (3) Ply1228, a lysin derived from *S. suis* SS12, acts against eight serotypes of *S. suis* (22); and (4) the phage-encoded lytic enzyme LySMP, recognized for its potency, is limited to serotypes 2, 7, and 9 and exhibits relatively low efficiency. It reduces turbidity by only 30% or even less in most strains after a 30-min exposure (22). Conversely, Ply691 acts swiftly, achieving its maximum bactericidal effect within just 20 min when interacting with *S. suis* SC267, with no significant change in efficiency upon prolonged exposure. This rapid and broad-spectrum activity underscores Ply691’s potential as a highly effective lysin.

The modest *in vitro* reduction (≈1 log CFU) is consistent with several phage lysins. The *S. aureus* lysin ClyS produced 0.8–1.3 log reductions in planktonic culture yet conferred full protection in murine bacteremia (23). Likewise, LysEF-P10 yielded ~1 log drop in vitro but cleared Enterococcus from the bloodstream. These data underscore that *in vivo* protection can be achieved even with limited planktonic killing because lysins act synergistically with host immunity and can target bacterial niches not reflected in broth assays (24).

In mouse infection studies with *S. suis*, 2 mg of Ply30 protected 90% of the mice (9) and PlySs2 shielded 92% (21). Instead, a 2 mg dose of Ply691 effectively prevented mortality in all bacteremic mice. This underscores Ply691’s potential as a highly effective therapeutic agent. Safety assessments confirmed Ply691’s non-toxicity to mice while maintaining its efficacy.

Another important aspect is that *S. suis* SC267 did not develop resistance to Ply691, neither *in-vivo* nor *in-vitro*, during the tested time. This resistance profile aligns with other lysins, such as the staphylococcal chimeric lysin ClyS (23), *S. aureus* phage lysin LysGH15 (20), and *E. faecalis* phage lysin LysEF-P10 (24). These findings reinforce the general mechanism of lysins, indicating that Ply691 holds considerable promise for clinical therapeutic applications.

As with other lysins, Ply691’s proteinaceous nature raises immunogenicity concerns; repeated administration of bacteriophage lysins has induced neutralizing antibody responses in animal models, potentially reducing efficacy over time (25). Delivery limitations persist: oral administration is hindered by gastric proteolysis, necessitating parenteral routes or protective formulations such as pH-sensitive hydrogels or nanoparticle encapsulation that have improved lysin stability in preclinical studies (26). Large-scale production costs and shelf-life stability under clinical storage conditions also require optimization, as reported for other therapeutic lysins (27). Addressing these hurdles will be critical for advancing Ply691 toward clinical application.

## 5 Conclusion

This study demonstrates the successful expression of *S. suis* bacteriophage lysin Ply691 in *E. coli*, with recombinant Ply691 exhibiting broad-spectrum lytic activity against 11 serotypes. Crucially, Ply691 maintains significant stability across clinically relevant conditions (4 °C–37 °C; pH 7–10) and shows potent therapeutic efficacy in murine bacteremia models, substantially reducing bacterial load. However, the prokaryotic expression system potentially limits accurate functional assessment due to absent post-translational modifications, while therapeutic validation remains confined to bacteremia models without exploration of meningitis or pneumonia contexts. Future work should prioritize: eukaryotic expression optimization to enhance biological relevance; validation in diverse infection models; and structure-guided protein engineering coupled with comprehensive stability profiling. Addressing these gaps will materially accelerate Ply691’s translational development toward clinical therapeutic applications.

## Data Availability

The datasets presented in this study can be found in online repositories. The nucleotide sequence of Ply267 has been deposited in the GenBank data base (https://www.ncbi.nlm.nih.gov/genbank/) under accession number PQ015119.

## Footnotes

^1^ http://pfam.xfam.org/

^2^ https://www.ncbi.nlm.nih.gov/Structure/cdd/wrpsb.cgi

## References

[1] Hayer SS Rovira A Olsen K Johnson TJ Vannucci F Rendahl A. Prevalence and time trend analysis of antimicrobial resistance in respiratory bacterial pathogens collected from diseased pigs in USA between 2006-2016. Res Vet Sci. (2020) 128:135–44. doi: 10.1016/j.rvsc.2019.11.010, PMID: 31785428

[2] Feng Y Zhang H Ma Y Gao GF. Uncovering newly emerging variants of *Streptococcus suis*, an important zoonotic agent. Trends Microbiol. (2010) 18:124–31. doi: 10.1016/j.tim.2009.12.003, PMID: 20071175

[3] Briers Y Walmagh M Van Puyenbroeck V Cornelissen A Cenens W Aertsen A. Engineered endolysin-based "Artilysins" to combat multidrug-resistant gram-negative pathogens. MBio. (2014) 5:e01379–14. doi: 10.1128/mBio.01379-14, PMID: 24987094 PMC4161244

[4] Fischetti VA. Bacteriophage endolysins: a novel anti-infective to control gram-positive pathogens. Int J Med Microbiol. (2010) 300:357–62. doi: 10.1016/j.ijmm.2010.04.002, PMID: 20452280 PMC3666336

[5] Spratt BG. Resistance to antibiotics mediated by target alterations. Science. (1994) 264:388–93. doi: 10.1126/science.8153626, PMID: 8153626

[6] Fischetti VA. Bacteriophage lytic enzymes: novel anti-infectives. Trends Microbiol. (2005) 13:491–6. doi: 10.1016/j.tim.2005.08.007, PMID: 16125935

[7] Ma YL Lu CP. Isolation and identification of a bacteriophage capable of infecting *Streptococcus suis* type 2 strains. Vet Microbiol. (2008) 132:340–7. doi: 10.1016/j.vetmic.2008.05.013, PMID: 18676101

[8] Gilmer DB Schmitz JE Euler CW Fischetti VA. Novel bacteriophage lysin with broad lytic activity protects against mixed infection by *Streptococcus pyogenes* and methicillin-resistant *Staphylococcus aureus*. Antimicrob Agents Chemother. (2013) 57:2743–50. doi: 10.1128/AAC.02526-12, PMID: 23571534 PMC3716137

[9] Tang F Li D Wang H Ma Z Lu C Dai J. Prophage lysin ply 30 protects mice from *Streptococcus suis* and *Streptococcus equi* subsp. zooepidemicus infections. Appl Environ Microbiol. (2015) 81:7377–84. doi: 10.1128/AEM.02300-15, PMID: 26253669 PMC4592866

[10] Ji W Huang Q Sun L Wang H Yan Y Sun J. A novel endolysin disrupts *Streptococcus suis* with high efficiency. FEMS Microbiol Lett. (2015) 362:fnv 205. doi: 10.1093/femsle/fnv205, PMID: 26534896

[11] Zhang H Zhang C Wang H Yan YX Sun J. A novel prophage lysin ply 5218 with extended lytic activity and stability against *Streptococcus suis* infection. FEMS Microbiol Lett. (2016) 363:fnw 186. doi: 10.1093/femsle/fnw186, PMID: 27481700

[12] Wang Z Liu X Shi Z Zhao R Ji Y Tang F. A novel lysin ply 1228 provides efficient protection against *Streptococcus suis* type 2 infection in a murine bacteremia model. Vet Microbiol. (2022) 268:109425. doi: 10.1016/j.vetmic.2022.109425, PMID: 35397385

[13] Wang S Li X Ma J Duan X Wang H Wang L. Structural and functional analysis reveals the catalytic mechanism and substrate binding mode of the broad-spectrum endolysin ply 2741. Virulence. (2025) 16:2449025. doi: 10.1080/21505594.2024.2449025, PMID: 39810299 PMC11740692

[14] Vouillamoz J Entenza JM Giddey M Fischetti VA Moreillon P Resch G. Bactericidal synergism between daptomycin and the phage lysin Cpl-1 in a mouse model of pneumococcal bacteraemia. Int J Antimicrob Agents. (2013) 42:416–21. doi: 10.1016/j.ijantimicag.2013.06.020 23992647

[15] Xi H Fu Y Chen C Feng X Han W Gu J. *Aerococcus viridans* phage Lysin AVPL had lytic activity against *Streptococcus suis* in a mouse bacteremia model. Int J Mol Sci. (2023) 24:16670. doi: 10.3390/ijms242316670, PMID: 38068990 PMC10706753

[16] Bateman A Rawlings ND. The CHAP domain: a large family of amidases including GSP amidase and peptidoglycan hydrolases. Trends Biochem Sci. (2003) 28:234–7. doi: 10.1016/S0968-0004(03)00061-6, PMID: 12765834

[17] Rigden DJ Jedrzejas MJ Galperin MY. Amidase domains from bacterial and phage autolysins define a family of gamma-D, l-glutamate-specific amidohydrolases. Trends Biochem Sci. (2003) 28:230–4. doi: 10.1016/s0968-0004(03)00062-8, PMID: 12765833

[18] Obeso JM Martínez B Rodríguez A García P. Lytic activity of the recombinant staphylococcal bacteriophage phi H5 endolysin active against *Staphylococcus aureus* in milk. Int J Food Microbiol. (2008) 128:212–8. doi: 10.1016/j.ijfoodmicro.2008.08.010, PMID: 18809219

[19] Huang Y Yang H Yu J Wei H. Molecular dissection of phage lysin ply Ss2: integrity of the catalytic and cell wall binding domains is essential for its broad lytic activity. Virol Sin. (2015) 30:45–51. doi: 10.1007/s12250-014-3535-6, PMID: 25680444 PMC8200883

[20] Zhang L Li D Li X Hu L Cheng M Xia F. Lys GH15 kills *Staphylococcus aureus* without being affected by the humoral immune response or inducing inflammation. Sci Rep. (2016) 6:29344. doi: 10.1038/srep29344, PMID: 27385518 PMC4935890

[21] Gilmer DB Schmitz JE Thandar M Euler CW Fischetti VA. The phage lysin ply Ss2 decolonizes *Streptococcus suis* from murine intranasal mucosa. PLoS One. (2017) 12:e0169180. doi: 10.1371/journal.pone.0169180, PMID: 28046082 PMC5207509

[22] Wang Y Sun JH Lu CP. Purified recombinant phage lysin LySMP: an extensive spectrum of lytic activity for swine streptococci. Curr Microbiol. (2009) 58:609–15. doi: 10.1007/s00284-009-9379-x, PMID: 19267155

[23] Pastagia M Euler C Chahales P Fuentes-Duculan J Krueger JG Fischetti VA. A novel chimeric lysin shows superiority to mupirocin for skin decolonization of methicillin-resistant and-sensitive *Staphylococcus aureus* strains. Antimicrob Agents Chemother. (2011) 55:738–44. doi: 10.1128/AAC.00890-10, PMID: 21098252 PMC3028755

[24] Cheng M Liang J Zhang Y Hu L Gong P Cai R. The bacteriophage EF-P 29 efficiently protects against lethal vancomycin-resistant *Enterococcus faecalis* and alleviates gut microbiota imbalance in a murine bacteremia model. Front Microbiol. (2017) 8:837. doi: 10.3389/fmicb.2017.00837, PMID: 28536572 PMC5423268

[25] Zhao M Huang M Li Z. Exploring the therapeutic potential of recombinant human lysozyme: a review on wound management system with antibacterial. Front Bioeng Biotechnol. (2023) 11:1292149. doi: 10.3389/fbioe.2023.1292149, PMID: 38026866 PMC10646323

[26] Wang Y Lv Q Chen Y Xu L Feng M Xiong Z. Bilayer hydrogel dressing with lysozyme-enhanced photothermal therapy for biofilm eradication and accelerated chronic wound repair. Acta Pharm Sin B. (2023) 13:284–97. doi: 10.1016/j.apsb.2022.03.024, PMID: 36811095 PMC9939289

[27] Murray E Draper LA Ross RP Hill C. The advantages and challenges of using Endolysins in a clinical setting. Viruses. (2021) 13:680. doi: 10.3390/v13040680, PMID: 33920965 PMC8071259

